# Hemosiderin deposition evaluation in hemophilic ankle joints: association between US finding and gradient-recalled echo MR imaging sequence

**DOI:** 10.1186/s13244-021-01050-1

**Published:** 2021-07-27

**Authors:** Marcel Prasetyo, Ariel Elisa Mongan, Novie Amelia Chozie, Joedo Prihartono, Stefanus Imanuel Setiawan

**Affiliations:** 1 Department of Radiology, Faculty of Medicine, Universitas Indonesia – Dr. Cipto Mangunkusumo National Central General Hospital, Jl. Diponegoro No.71, Jakarta Pusat, DKI Jakarta 10430 Indonesia; 2 Department of Pediatric, Faculty of Medicine, Universitas Indonesia – Dr. Cipto Mangunkusumo National Central General Hospital, Jakarta, Indonesia; 3 Departement of Community Medicine, Faculty of Medicine, Universitas Indonesia, Jakarta, Indonesia

**Keywords:** Hemosiderin deposition, Hemophilic arthropathy, US, GRE MR imaging

## Abstract

**Background:**

Repeated bleeding in hemophilic arthropathy (HA) may result in severe degenerative changes and joint destruction. The gradient-recalled echo (GRE) sequence MR is proved to be the best method to detect hemosiderin deposition. However, MR is not widely available in developing countries, including Indonesia. Some studies have proposed ultrasonography (US) as an alternative tool in evaluating hemophilic joint. However, there is still some disagreement on the ability of US to detect hemosiderin deposition.

**Objective:**

To evaluate the association between US and GRE-sequence MR imaging in detecting hemosiderin deposition in hemophilic ankle joint.

**Material and methods:**

A total of 102 sites from 17 ankle joints of 11 boys with severe hemophilia A underwent US examination using a high-frequency linear array transducer. GRE-sequence MR examination was performed in sagittal view consistent with the sites scanned by US. Both examinations were performed on the same day, but MR interpretation was performed blindly at different times. The association between US and GRE-sequences in detecting hemosiderin deposition was analyzed using McNemar’s test.

**Results:**

Statistical analysis showed a significant association (*p* value < 0.001) between US and GRE MR in detecting hemosiderin deposition, but the association is weak (*R* = 0.26). Sensitivity and specificity of US for detecting hemosiderin deposition were 46.84% (95%CI: 35.51–58.40) and 95.65% (95%CI: 78.05–99.89), respectively, with positive predictive value 97.37% (95%CI: 84.29–99.61), negative predictive value 34.38% (95%CI: 29.50–39.60) and accuracy 57.84% (95%CI: 47.66–67.56).

**Conclusion:**

There was a weak association between US and GRE-sequences in detecting hemosiderin deposition of hemophilic ankle joint. ​​

## Key points

- US can evaluate hemosiderin better if there are a large amount and heterogeneous echotexture of hemosiderin deposition.
- There is a weak association between US and GRE MR in detecting hemosiderin deposition.
- Hemosiderin deposition could be easier to be identified in the anterior and posterior recess of ankle joint.

## Introduction

Hemophilic arthropathy (HA) is the most common clinical manifestation of hemophilia. Recurrent bleeding causes synovial hypertrophy, hemosiderin deposition, cartilage destruction, and changes in the structure of subchondral bone. Hemosiderin deposition can stimulate further synovial hypertrophy and inflammation. As a result, joints become painful and could lose their physiological function which further potentially causes disabilities [1–4]. Magnetic Resonance (MR) imaging is superior to other imaging modality in detecting soft tissue changes, such as synovial hypertrophy, hemarthrosis, joint effusion, cartilage defect, and changes in bone structure [5–7]. The gradient-recalled echo (GRE) MR imaging sequence is regarded as a sensitive method in evaluating hemosiderin deposition [8]. However, MR imaging is not the first choice of imaging considering the cost, need of sedation, time-consuming, limited access in developing countries, and inability to perform imaging in multiple joints at the same time [9].

Many studies have proposed the use of US in evaluating HA [4–8, 10–17]. However, there was some disagreement regarding US ability in detecting hemosiderin deposition in hemophilic joint. Doria et al. [14] reported the sensitivity and specificity of US for hemosiderin detection are 100% and 67%, respectively, and it was shown as hypoechoic structures. Hemosiderin deposition outside of the synovium is also reported. According to Martinoli et al. [15], hemosiderin is embedded inside the synovium and does not accumulate in joint space which makes misleading interpretation of US findings on hemosiderin deposition. Different opinions on the echogenicity of hemosiderin are also noted, as Melchiorre et al. [4] defined hemosiderin deposition as a diffuse hyperechoic area. This study aims to evaluate US ability to identify hemosiderin deposition at the same anatomical sites of hemophilic ankle joint, in comparison with GRE-sequence MR imaging.

## Materials and methods

**Fig. 1:**
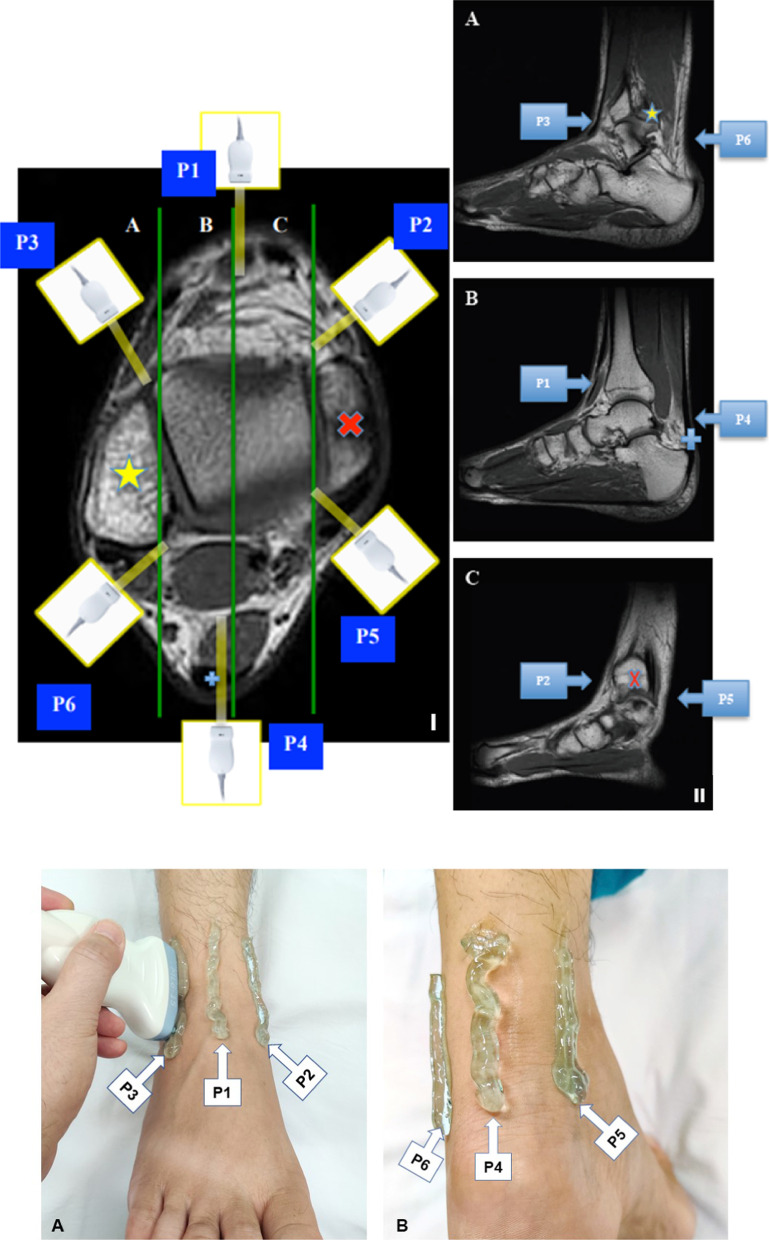
Sites observed for each ankle. **i** Axial view; **ii** sagittal view; **a** anterior approach; **b** posterior approach: mid ankle (P1), anterior to medial malleolus (P2), anterior to the lateral malleolus (P3), midline (P4), posterior to medial malleolus (P5), and posterior to the lateral malleolus (P6)

A cross-sectional study was performed on ankle joints of boys aged 7–18 years old with severe hemophilia A and no history of inhibitor. Ankle joints examined were considered as target joint with HEAD-US score ≥ 6. Each child participant’s guardian was given written informed consent and the patient data were kept anonymous and confidential. This study was conducted in the Cipto Mangunkusumo National General Hospital (RSUPN-CM), Jakarta. Ankle US was performed in 6 sites, based on the method proposed by Zukotynski et al. [16] with some modification. In this study, the 6 sites were marked as P1–P6. Anterior central scan (P1) was performed with transducer placed above middle area of the ankle and parallel to the long axis of the leg, anteromedial (P2) landmark was anterior aspect of medial malleolus, and anterolateral (P3) landmark was anterior aspect of lateral malleolus. Anterior scan (P1-P3) was performed with subject in supine position, while posterior scan (P4-P6) in prone position. The posterior central scan (P4) was performed when transducer was placed above the Achilles tendon parallel to tendon axis, posteromedial (P5) landmark was posterior aspect of medial malleolus, and posterolateral (P6) landmark was posterior aspect of lateral malleolus (Fig. 1). Subjects with ankle joint deformity and history of synovectomy were excluded. Ankle US was performed in longitudinal view on the P1–P6 locations, using Philips Affinity 70® US machine with a 12-MHz linear array transducer. Positive US finding for hemosiderin is defined as a hypoechoic structure with indistinct border, either within the synovial cavity or embedded within synovial wall.

Ankle MR was performed using 1.5 Tesla GE Optima® machine. Patient was positioned in supine position, and the ankle was scanned using dedicated RF coil for ankle with the single-channel 1.5 HD T/R Quad Extremity coil. Sequence performed is gradient-recalled echo T2* in true sagittal section; repetition time 674 ms, echo time 14,5 ms, flip angle 30°, slice thickness 4 mm, a field of view 18 cm, matrix 256 × 192. Besides, T2 fat suppression routine sequence in the sagittal section was also performed. Positive MR finding for hemosiderin is defined as a hypointense blooming artifact on GRE sequence, located at the P1-P6 sites consistent with US scan locations.

US and MR scans were performed on the same day. US results were interpreted directly during scanning, while MR results were interpreted blindly approximately 3 weeks after scanning. US and MR interpretation were performed by a musculoskeletal radiologist with at least 10-years of experience in musculoskeletal ultrasound, who was also a member of Multidisciplinary Hemophilia Management Team of Cipto Mangunkusumo General Hospital, Jakarta. False negative echogenicity features of US examination, including isoechoic and hyperechoic findings, but positive minimal hemosiderin deposition in MR were recorded descriptively and further discussed. Statistical analysis using McNemar’s test was performed using SPSS 20 to measure the association in detecting hemosiderin between US and MR. Sensitivity and specificity analyses were also done to assess the capability of US in detecting hemosiderin compared to MR. Cohen’s Kappa R value was also calculated to show the concordance between US and MR findings. The Kappa R value was further categorized into > 0.75 as ‘strong interobserver agreement’; 0.4–0.75 as ‘moderate interobserver agreement’; < 0.4 as ‘weak interobserver agreement.’

## Results

A total of 102 sites from 17 ankle joints obtained from 11 boys with characteristics described further in Table 1. US and MR showed a weak association in detecting hemosiderin (*R* = 0.26, *p* < 0.001) (Table 2). Sensitivity and specificity of US for detecting hemosiderin deposition were 46.84% (95%CI: 35.51–58.40) and 95.65% (95%CI: 78.05–99.89), respectively, with positive predictive value 97.37% (95%CI: 84.29–99.61), negative predictive value 34.38% (95%CI: 29.50–39.60) and accuracy 57.84% (95%CI: 47.66–67.56).

**Table 1:** Characteristics of subjects

Subject characteristics	*N* (%)
*Age groups*	
< 10 years old	4 (36.4)
> 10 years old	7 (63.6)
*Affected ankle joint*	
Unilateral (right ankle joint)	3 (27.3)
Unilateral (left ankle joint)	2 (18.2)
Bilateral	6 (36.4)
*HEAD-US score of the right ankle joint*	
HEAD-US score: 6	4 (36.4)
HEAD-US score: 7	3 (27.3)
Missing	4 (36.4)
*HEAD-US score of the left ankle joint*	
HEAD-US score: 6	2 (18.2)
HEAD-US score: 7	4 (36.4)
HEAD-US score: 8	2 (18.2)
Missing	3 (27.3)

**Table 2:** Association between US and MRI results in detecting hemosiderin deposition

Hemosiderin deposition finding in the US	Hemosiderin deposition in the GRE MR imaging sequences		Total *N*	*p* value
	Positive *N* (%)	Negative *N* (%)		
Positive	37 (97.37)	1 (2.63)	38	< 0.05
Negative	42 (65.63)	22 (34.37)	64	
Total	79 (77.45)	23 (22.55)	102	

**Fig. 2:**
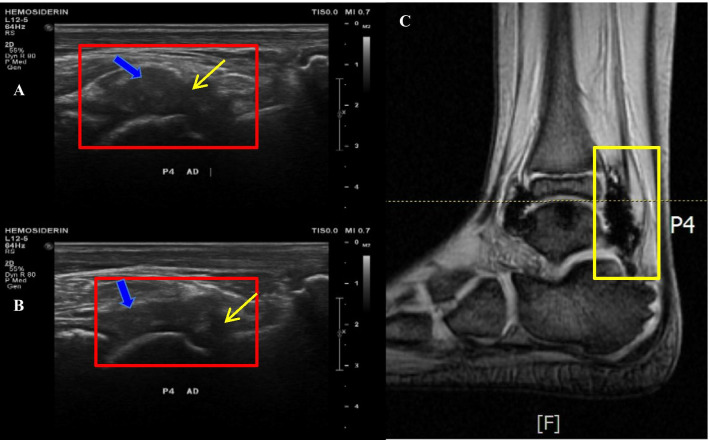
**a** US finding in P4 location on right ankle shows the hypoechoic area (thin yellow arrow) with hypertrophic synovium with the intermediate-echogenicity area (thick blue arrow) that was moderately compressible (**b**). **c** The MR study in the same location confirms hemosiderin

**Fig. 3:**
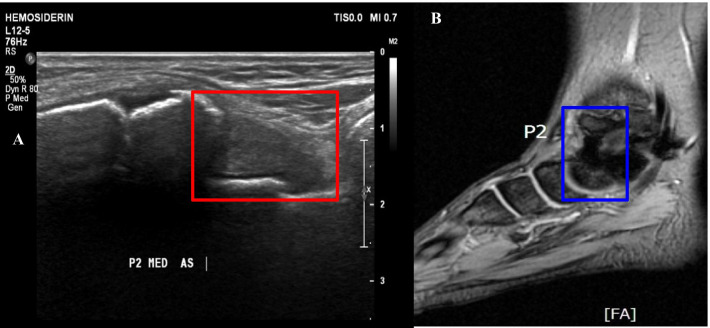
**a** US finding in P2 location (anterior-medial) shows a hypoechoic area (red box) inferior to the medial malleolus, consistent with hemosiderin on MR comparison (**b**)

Similar locations of hemosiderin found on US and MR are following: 12 (32.43%) samples in P4 (Fig. 2); 9 (24.32%) samples in P1; 5 (13.51%) samples in P3, P5 and P6; and 1 (2.70%) sample in P2 (Fig. 3). Negative US findings but positive MR were found in following locations: 12 (28.57%) samples in P2, 9 (21.43%) samples in P6, 8 (19.05%) samples in P3, 7 (16.67%) samples in P5, 5 (11.90%) samples in P1, and 1 (2.38%) sample in P4.

Based on positive MR of hemosiderin deposition (*n* = 79), US echogenicity was mostly hypoechoic on 73 samples (92.0%), while only 4 samples (5.0%) were found isoechoic and 2 samples (3.0%) hyperechoic.

## Discussion

There was a statistically significant difference in results between US and MR imaging in detecting hemosiderin deposition in hemophilic joints and the concordance value is almost equal with discordance value. US also has low sensitivity with relatively low negative predictive value in detecting hemosiderin deposition, resulting from three main factors: the amount of hemosiderin, the echotexture of hemosiderin, and the location of sampling. These three factors were based on descriptive data distribution found in this study. Non-corresponding data between MR and US were found. MR could detect minimal hemosiderin observed as dots, which is not detectable and hard to be visualized by US. This minimal hemosiderin deposition could be easily found in MR imaging, but in US, it is difficult to be differentiated with the surrounding structures such as synovial thickening, cartilage, and fat pads which lead to misinterpretation of the structure as non-hemosiderin, similar to previous studies by Martinoli et al*.* [15], Zukotynski et al*.* [16] and Prasetyo et al*.* [17].

The second factor is the echotexture phenomenon in this study. Hemosiderin echotexture distribution was dominant to be heterogeneous echotexture. In this study, homogenous hypoechoic echotexture finding was mostly not associated with MR results due to its similarity to synovial thickening, while the heterogeneous hypoechoic echotexture finding tends to associate with MR results. Based on these results, in an area with relatively higher echogenicity, it is observed that hemosiderin deposition which characterized by a hypoechoic area could create a heterogeneous characteristic. A study by Doria et al. [14] also found that synovium has a wide echotexture and showed a varying level of hemosiderin deposition. However, that study demonstrated a high sensitivity (> 92%) of US in detecting hemosiderin deposition compared to MR findings, a much higher sensitivity compared to this study (46.84% (95%CI: 35.51–58.40)). This could be explained by the methodology used in this study which compared data from MR and US at the same anatomical level, not as a whole ankle. Furthermore, the number of joint bleeding and a history of bleeding recurrences can affect the echogenicity of hemosiderin deposition.

The third factor that affected hemosiderin detection in US is the sampling location. The location with the highest positive results in detecting hemosiderin deposition using both US and MR were P4 location (central-posterior) and P1 (central-anterior) since these locations are sensitive in detecting joint effusion fluid, which could push a high amount of fat pad. [18] Furthermore, the probe could be easily placed parallel to long axis of tibial bone (anterior side) and calcaneal tendon (posterior side), thus creating minimal artifact and anisotropy. However, the bulging of malleolus and the different size of each patient’s ankle, to assess the lateral and medial part, were causing probe surface to not fully adhere to the skin surface. This factor caused the observed area to not be optimally visualized, especially in deeper areas_,_ which created shadow refraction artifacts and anisotropy. These technical issues need to be considered to minimalize artifacts which would later affect the detection and identification of hemosiderin deposition and may be prevented by using more gel on the gap between the probe and skin or using a smaller probe (hockey stick).

All positive joint hemosiderin deposition on both modalities was accompanied by severe synovial hypertrophy. This is related to HA pathophysiology and cycle, the process of hemarthrosis-synovitis-hemarthrosis, and could be concluded that hemosiderin tends to be detected at conditions with severe synovial thickening [3].

**Fig. 4:**
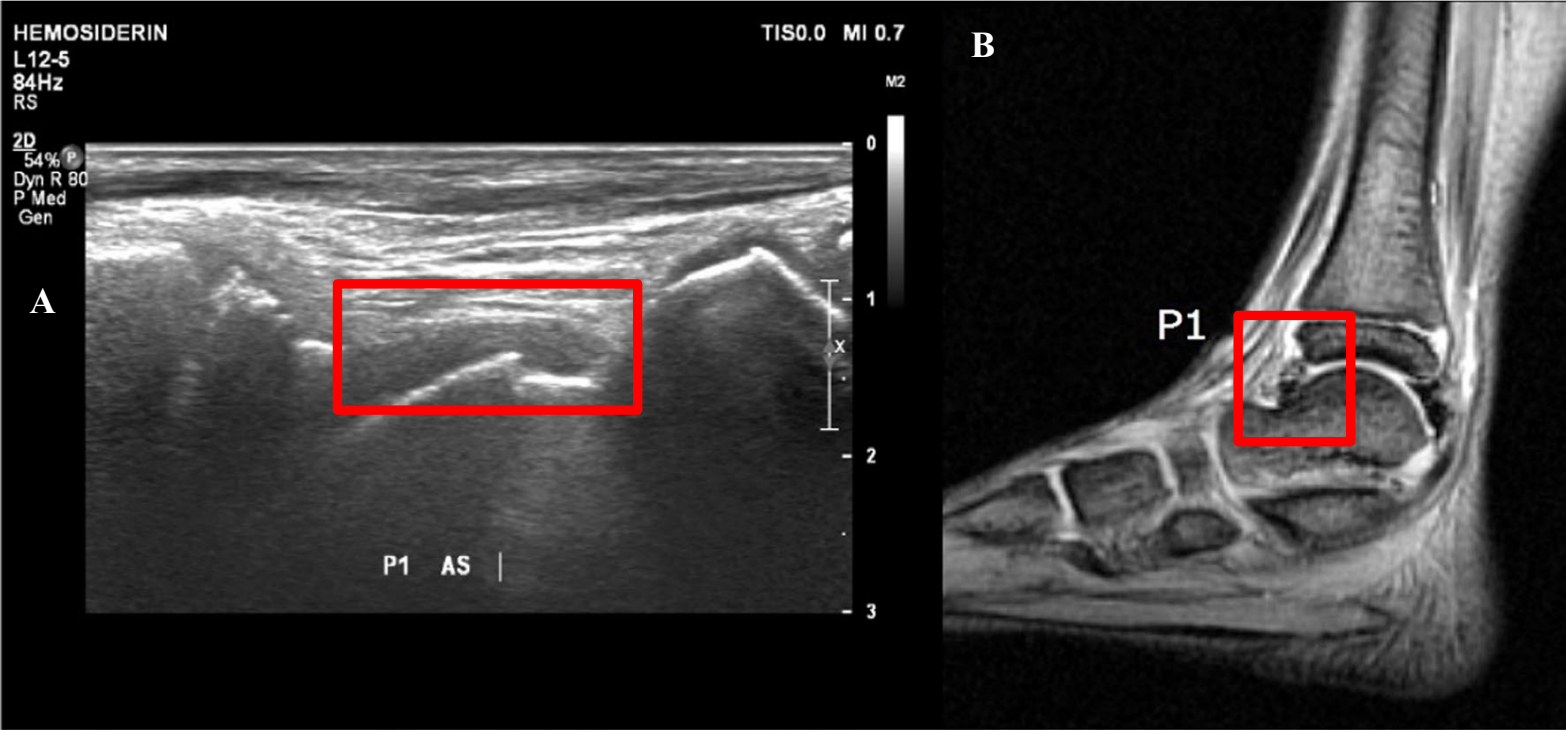
US examination at P1 location on the left ankle (**a**) showed synovial thickening as confirmed by MRI (**b**). Minimal hemosiderin (red box) is difficult to visualize on the US due to its closed proximity to the thickened synovium

The hemosiderin deposition is mostly found as hypoechoic. Only a small number of samples were found to be isoechoic and hyperechoic shown in MR imaging as small focal spots of hemosiderin deposition (Fig. 4). These echogenicity features resulted as negative interpretation of hemosiderin deposition in US because they were hard to be differentiated from the surrounding structures. Thus, the ability of US in detecting hemosiderin might depend on the severity of hemosiderin content.

The limitation of this study is that hypoechoic structure seen in US as hemosiderin could not be confirmed directly because a histopathology examination was not performed. Besides, both US examination and the interpretation of the GRE sequences were performed by a single musculoskeletal radiologist. This study does not assess US performance to detect hemosiderin for one patient, but for one anatomical level, hence low sensitivity of ultrasound reported in this study. However, this study could highlight the best sagittal transducer positions for hemosiderin detection in ankle joint, which is anterior and posterior recess.

## Conclusion

There is a weak association between US and MR imaging in detecting hemosiderin deposition. The sensitivity and specificity of US for detecting hemosiderin deposition were 46.8% and 95.7%, respectively, with the accuracy of 57.8%. Hemosiderin deposition in US is characterized as a hypoechoic structure relative to the synovium, which is easier to be identified in thick hypertrophic synovium, especially in the anterior and posterior recess of ankle joint.

## Data Availability

Supporting data were available for review by the corresponding author.

## Abbreviations

GRE: Gradient-recalled echo
HA: Hemophilic arthropathy
HEAD-US: Hemophilia Early Arthropathy Detection with Ultrasound
MR: Magnetic Resonance
US: Ultrasonography
